# Indigenous Health and Human Rights: A Reflection on Law and Culture

**DOI:** 10.3390/ijerph15040789

**Published:** 2018-04-18

**Authors:** Odette Mazel

**Affiliations:** Melbourne Law School and Faculty of Medicine, Dentistry and Health Sciences, The University of Melbourne, Carlton 3010, Australia; omazel@unimelb.edu.au; Tel.: +61-3-8344-9160

**Keywords:** Indigenous health, human rights, postcolonial theory

## Abstract

In Australia, Aboriginal and Torres Strait Islander peoples bear a greater burden of disease and have lower life expectancy than their non-Indigenous counterparts. These combined indicators are evidence of an entrenched health crisis in the Indigenous population that is linked to systemic disadvantage over many decades. In an effort to improve life expectancy and lessen the burden of disease, a number of strategies and national frameworks now embed a human rights-based approach to achieving health equality. This paper explores the application of human rights to Indigenous health and examines the inherent tensions that exist in engaging a system of law based on universal assumptions of the Enlightenment to advance Indigenous rights. What becomes apparent through this exploration is that the strategic approach of Indigenous peoples’ use of human rights, despite its genesis in a system of law that justified colonisation, has opened up opportunities to reframe fixed ideas of law and culture.

## 1. Introduction

*(Indigenous peoples have survived with) great resilience in the face of tremendous adversity… they have survived as they have striven to maintain the cultural integrity that makes them different, while adapting, often ingeniously, to the changing conditions around them.*

*[1] (p. 13)*

Aboriginal and Torres Strait Islanders in Australia continue to experience significant health, economic and social disadvantage [2]. In an effort to improve life expectancy and lessen the burden of disease, a number of strategies and national frameworks have embedded a human rights-based approach to achieving health equality. Despite its genesis in a system of law that justified colonisation and the disenfranchisement of Indigenous peoples, human rights have emerged as the international language through which resistance from oppression has been expressed, and an instrument by which liberation can, theoretically, be achieved. In this paper, I examine the inherent tensions that exist in engaging with a system of law based on universal assumptions of the Enlightenment to advance Indigenous rights and demonstrate how Aboriginal and Torres Strait Islander people have made use of, as well as influenced, international human rights discourse and law in their effort to combat systemic health and socioeconomic disadvantage. While there is still a way to go along a complex path before Aboriginal and Torres Strait Islander people achieve health equality, the human rights framework has provided Indigenous Australians with tools to influence government strategies concerning health. At the same time, Aboriginal and Torres Strait Islander social movements have, themselves, shaped and informed the design and use in practice of human rights. This exchange is illustrative, not of a resignation to a dominant system of law, but a counter-hegemonic challenge in which resistant practices are transforming the nature and operation of the law.

I begin in Section 2 by exploring the genesis of the human rights framework and the implicit risk of re-inscribing or reiterating neo-colonial imperatives. In Section 3, I explore the postcolonial discourses that underpin theories on difference to demonstrate the strategic thinking behind Indigenous peoples’ use of human rights and how this seemingly discordant pairing might open up opportunities to reframe fixed ideas of law and culture. In Section 4, I examine and critique the application of human rights principles in Australian policy initiatives relating to Aboriginal and Torres Strait Islander health. 

## 2. Forging an Indigenous Presence in International Human Rights Law

In this section, I examine the historical effects of colonisation on Aboriginal and Torres Strait Islanders and the poor health outcomes that have resulted from decades of disenfranchisement. I then look at the role of law in the colonisation process, engaging theories of difference to understand how Indigenous peoples in Australia were socially, culturally and politically marginalised, as well as the role law played in inspiring the Indigenous activism of the 1950s and 1960s, which set the scene for a social movement that sparked a number of advances in Indigenous affairs. Finally, I look at the development of international human rights law and discourse in regards to Indigenous peoples and the ways in which international law is adapting to this particular site of difference with the adoption of the UN Declaration on the Rights of Indigenous Peoples (UNDRIP) in 2007.

### 2.1. Health Status

The Australian Bureau of Statistics estimates that the life expectancy gap between Indigenous and other Australians is 10.6 years for males and 9.5 years for females [3]. Indigenous Australians bear a disproportionate burden of disease at a rate 2.5 times that of a non-Indigenous person [4]. Indigenous Australians are 5 times more likely to suffer from diabetes, 4.5 times more susceptible to cardiovascular disease, and 4 times more prone to injuries from violence [5] (p. 1). At every age, Indigenous Australians are sicker, and die earlier, than their non-Indigenous counterparts [4,6]. While there has been some improvement in the rates of Indigenous infant and child mortality, the incidence of diabetes, mental illness, heart disease and smoking-related cancers is increasing [5,7]. The pattern of health disadvantage is echoed across a range of other social indicators such as employment, income, education and housing [8] (p. 761). On average, Indigenous Australians have considerably lower incomes, higher rates of unemployment, lower educational attainment and more overcrowded households [9]. They are also more likely to be imprisoned, suffer from childhood abuse and violence and are at greater risk of suicide [9] (pp. 4–6). This socioeconomic disadvantage exposes Indigenous Australians to higher levels of unhealthy risk factors and contributes to the increased experience of lateral violence—the result of historical power imbalances—continuing negative stereotypes and trauma [10,11]. These combined indicators are evidence of an entrenched health crisis in the Indigenous population that is linked to colonisation and systemic disadvantage experienced over many decades [12] (p. 22).

Since colonisation, Aboriginal and Torres Strait Islanders have been subjected to intense levels of discrimination that have involved the dispossession of their law, lands, languages and economic systems, and have decimated the population through disease and brute force [13] (p. 82). Indigenous peoples were forced onto missions and subjected to laws that legitimated the forceful removal of their children. In every way, their culture was denied, and their way of life compromised in the name of “civilisation”. It was not until 1962 that Aboriginal people were able to vote in Commonwealth elections (although it was an offence to encourage it) [14], and it was not until the national referendum of 1967 that the race clause was removed from the Australian Constitution, marking the beginning of citizenship rights for Indigenous Australians [15]. These advances were the result of an Indigenous-led social movement, which emerged during the 1950s and 1960s, that fought fiercely for Aboriginal self-determination, sovereignty and community control [8]. Ian Anderson describes it as a proud and defiant “social movement that reclaimed a public space and inserted new forms of representation about (Indigenous) identities and cultures” that challenged and reshaped the mainstream view of Indigenous peoples as “bewildered remnants of a primitive and savage race [8] (p. 761)”. In the absence of a treaty or constitutional recognition, the rights-based approach of the still embryonic international human rights framework was used to mobilise support for the cause. This was an important political and philosophical partnership that eventually culminated in land rights legislation, the recognition of native title, and community control of services such as health services [8] (p. 760), [16] (p. 1777).

While there are obvious synergies between the Indigenous and human rights movements, there are also risks attached to the Indigenous movement relying too closely on the international human rights framework. International law was initially conceived of and shaped by humanitarian thinkers and legal practitioners of the dominant imperial powers of 18th and 19th centuries and, as a result, the human rights system of law and its philosophical principles are firmly underpinned by the liberal assumptions of the time, including the imperial narrative of the “civilising mission”. Theories about how difference was construed as “otherness”, dividing the European and non-European worlds, provide insight into this historical context and the role law played in legitimising the colonial project.

### 2.2. Law and Culture: The Civilising Mission

In order to understand the use of the human rights framework as a tool to support Indigenous health initiatives, it is important to understand the historical context of human rights at international law. “Knowing about the terrain”, claims Harraway, “… is a prerequisite for remapping the possible ground for new stories [17] (p. 295)”. The relationship between international law and Indigenous peoples is complex and ever changing. In the 16th century, negotiation of the legal and moral relationship between European states and Indigenous peoples marked the jurisprudential beginnings of the rights of peoples and the birth of international law. In the early 1500s, the Spanish philosopher and theologian Francisco de Vitoria [18], and those associated with the “Spanish School”, became concerned with the rights of Indians under the rule of the Spanish conquistadores in South America, and argued successfully for divine law, administered by the Pope, to be replaced by a universal natural law system whose rules were ascertained through the use of reason [19] (pp. 17–18). In so doing, Vitoria commenced his “construction of a new jurisprudence” in which natural law, administered by a secular sovereign, became the source of international law governing Spanish-Indian relations [19] (pp. 17–18). By acknowledging the Indians as having “reason”, they were understood to be a self-determining community who held rights under, and were subject to, natural law [19] (p. 18), [20] (pp. 17–19). While his intentions were to legitimate Spanish incursions into Indian society, rights were, nevertheless, conferred on the Indians as a consequence of being human [19].

With the rise of legal positivism in the 18th century came a departure from the natural law framework and an emphasis on positivist notions of law and philosophy, with liberalism as the dominant frame of reference. International law became the realm of consenting sovereign states, and Indigenous peoples’ rights under natural law were progressively diminished and brought within the jurisdiction of the domestic law of the successor states of the colonial empires [21] (p. 26). Justified by the imperialist notion of European cultural superiority, the doctrine of *terra nullius* legitimised the acquisition of territory of Indigenous peoples, including Indigenous Australians, and set in motion the legal denial of their rights [22] (p. 236). The idea that cultural differences divided the European and non-European worlds defined the civilising mission, in which non-European societies were characterised as backward and primitive. Anthony Anghie describes this as the “dynamic of difference”: the process of creating a gap between two cultures, demarcating one as “universal” and civilised and the other as “particular” and uncivilised, and seeking to bridge the gap by normalising (civilising) the aberrant society [19] (p. 4). This dynamic, he argues, building on Edward Said’s work on Orientalism [23], was at the core of many of the central doctrines of international law in which colonised peoples were excluded from the realms of sovereignty, law and society [19] (p. 4) and constructed as something altogether homogeneous and “other” [23]. The ideology of difference constructs clear borders between groups, identifying characteristics that mark the superiority of one group against the inferior characteristics of the others [24] (p. 126). This logic of identity, as suggested by Iris Marion Young, “generates dichotomy rather than unity, dichotomies of what is included and what is excluded [24] (p. 126)”. The importance of this manoeuvre was that the re-entry of these societies into the sphere of law could only take place on terms that subordinated and disempowered them [19] (p. 66).

The discretion that states had in regards to the different and unequal treatment of people within their domestic jurisdiction came under scrutiny following the atrocities of the Second World War and the adoption of the Charter of the United Nations (UN Charter) in 1945 [25]. While the prevailing ideology remained the same, natural law’s moral concept of rights re-emerged as a proper subject of international law in which the welfare of human beings was to be valued and rights once again came to be understood as belonging to every individual as a consequence of being alive [22] (p. 237). Promoting fundamental freedoms for all became one of the main purposes of the United Nations (UN). The UN Charter opened the way for non-state participation in the deliberative processes of the UN and gave legitimacy to struggles against colonialism spurring “a new anti-colonial and anti-racist consciousness and discourse [25] (see arts 1, 2, 55, 56, 73), [26] (p. 60)”. The provisions of the UN Charter, and the recognition of the right to self-determination, enabled many former colonies to realise independence, but did not extend to Indigenous peoples within settler states [27], [28] (pp. 92–93).

The first global enumeration of human rights, to which all human beings are inherently entitled, was the Universal Declaration of Human Rights (UDHR), adopted by the UN General Assembly in 1948 [29]. The UDHR was an important milestone in international law and, since its adoption, there has been a steady growth of legal standards applicable to a government’s treatment of its own people, along with increasing pressure from human rights organisations and Indigenous movements for their domestic implementation [20] (p. 17). However, while this new legal regime represented an important shift towards allowing non-state actors a legitimate voice in international law, it also proffered a solution that was seen to be deeply embedded in an ethnocentrically Western legal framework. Scholars, such as William Edward Burghardt Du Bois, brought to the fore early concerns that these “universal” rights would, in reality, work to the benefit of some, not all, suggesting instead the development of case-specific human rights for those that experienced inequalities arising from race, class and gender [30]. Debate amongst anthropologists went a step further, with many claiming that human rights should be wholly rejected as they enumerated rights and freedoms as universal that were, in fact, “culturally, ideologically, and politically nonuniversal” and therefore not representative of, or applicable to, most of the world’s population [31] (p. 288), [32] (p. 542), [33]. These cultural relativists saw as essential the recognition of cultural difference and distinguished between individual and collective societies. They stood in opposition to universalists who valued the existence of inalienable individual human rights available to all for the very fact of being human [31]. The debates on universality versus cultural relativity continue to be relevant to our understanding of the paradox of rights discussed below. However, in more recent times, the debate has turned to focus on how we might think beyond this binary and to reflect on both culture and law not as static and unyielding, but as fluid, adaptive, dynamic and creative [34,35,36]. This approach has been underscored by the notion of agency in which human actors are seen to have the capacity to understand their social experience and devise ways of operating with autonomy even within coercive environments. In the following section, I will look at the role of agency in redefining the nature and application of human rights law as it relates to Indigenous peoples.

### 2.3. Culture and Law: The Role of Agency

Formal acknowledgment of Indigenous peoples’ particular and collective rights within the human rights framework has been incremental. Persistent advocacy from Indigenous peoples, however, has continued to expand the scope of human rights, resulting in the gradual moderation of the doctrine of sovereignty. As Bain Attwood contends, in the complex interrelations of power, domination and agency, “hegemony is never and can never be complete [37] (p. 146)”. Agency, coupled with exclusion, promotes reaction and innovation. This space of resistance, in turn, can be transformative of the very elements of constraint or domination. Sally Engle Merry uses the concept of cultural appropriation to describe this phenomenon in relation to culture and law, understanding that the law is itself cultural in nature. Whilst the term “cultural appropriation” has been used elsewhere to describe the inappropriate adoption of cultural elements of a minority culture by members of a dominant culture, Engle Merry’s hypothesis presents a different application of the term. Culture, she suggests, is integral to power and “forms construct hegemonic understandings as well as the counter hegemonies that challenge these understandings [38] (p. 579)”. Indigenous people’s use and influence on the development of human rights law illustrates one such counter-hegemonic challenge. The appropriation—in this case, of an imposed or alien legal system—can be seen as an act of resistance even though it appears to be a capitulation [38] (p. 599). Whilst it is important to properly understand the strength of prevailing colonial and neo-colonial ideologies, Indigenous peoples have fought for a place within the human rights framework and, in doing so, have (and are) redefining modes of thinking and practice to influence structural and practical change. 

In 1957, the International Labour Organisation Convention 107 on Indigenous and Tribal Populations became the first convention within the UN framework to deal exclusively with Indigenous peoples, promoting the right to economic and social equality [39]. Despite its assimilationist orientation, Convention 107 was significant as a first attempt to codify the international obligations of states in respect of Indigenous and tribal populations. A number of international human rights instruments followed that, to varying degrees, incorporated articles specifically applicable to Indigenous peoples. The International Covenant on Civil and Political Rights (ICCPR) recognised the right of all “peoples” to self-determination, embracing the concept of group or collective rights [40], and specified the right of persons belonging to ethnic, religious or linguistic minorities to enjoy their own culture [40,41,42,43]. The International Convention on Economic, Social and Cultural Rights (ICESCR) also recognised the right of peoples to self-determination [44] and promoted equal enjoyment of all economic, social and cultural rights, and the International Convention on the Elimination of Racial Discrimination (CERD) prohibited all forms of racial discrimination [45], including against Indigenous peoples [46]. These and other instruments [47,48,49] addressed areas of disadvantage including health, and guaranteed important rights, providing individuals the opportunity to claim the same rights as everyone else within the human rights framework. However, the case for recognition of specific collective rights for Indigenous peoples, with a unique set of shared experiences resulting from colonisation, was still to be made.

Driven by Indigenous peoples in collaboration with non-government organisations and human rights scholars, a number of international conferences and appeals took place in the 1970s that drew attention to the specialised rights of Indigenous peoples within international law. The Report on the Problem of Discrimination Against Indigenous Populations—prepared by UN Special Rapporteur José R. Martinez Cobo of Ecuador and written over 13 years between 1972 and 1986—contributed significantly to the recognition of the plight of Indigenous peoples as a separate and pressing international concern and was key to the development of the contemporary relationship between Indigenous peoples and the UN [22] (p. 239), [50]. The UN World Conference on Discrimination Against Indigenous Populations, convened in Geneva in 1977, was the first international conference on Indigenous issues [51]. Followed by the UN Conference on Indigenous Peoples and the Land in 1981 [52], these fora worked to increase the petitions from Indigenous peoples to UN human rights bodies and were integral to building a coordinated response to Indigenous peoples’ concerns. These events were set against the landmark decision in the Advisory Opinion of the International Court of Justice on the Western Sahara case, in which the application of the doctrine of *terra nullius* to land inhabited by Indigenous peoples was rejected and overturned [53].

All these advances paved the way for the establishment of the Working Group on Indigenous Populations by the Sub-Commission on the Prevention of Discrimination and Protection of Minorities in 1982 [54]. Its mandate was to review the development of human rights as they applied to Indigenous populations and to scope the need for specific standards [55] (p. 41). In an unprecedented move, the Working Group included Indigenous peoples and organisations in its debates and considerations and was open to contributions as to the wording and content of the Declaration on the rights of Indigenous peoples that they were drafting [24] (p. 63). In this way, Indigenous peoples became participants, as opposed to objects, in an extensive multilateral dialogue about their rights [21] (p. 56). The Permanent Forum on Indigenous Issues was later established by the UN Economic and Social Council to provide expert advice on Indigenous issues and “raise awareness and promote integration and coordination of activities related to Indigenous issues within the UN system [56]”. By placing the Forum on the same organisational level as the Human Rights Commission, the Economic and Social Council made it the highest-level body that can be established at the UN without constitutional reform. The establishment of a permanent body heralded recognition from UN member states that Indigenous peoples needed to participate in decision-making processes concerning standards designed to apply to them. Their inclusion within the UN structure meant Indigenous peoples were contributing as subjects and makers of international law [22] (p. 239).

As the united voice of Indigenous peoples across the globe grew in strength and influence, human rights law followed suit and in 1989 the International Labour Organisation Convention on Indigenous and Tribal Peoples, Convention No. 169, replaced Convention 107 rejecting assimilationism and defining rights more in line with Indigenous peoples’ objectives [57,58]. As stated in the preamble, Convention 169 aimed to recognise “the aspirations of (Indigenous) peoples to exercise control over their institutions, ways of life and economic development and to maintain and develop their identities, languages and religions [58]”. While Convention 169 remains one of the most significant legally binding documents in the human rights system for Indigenous peoples, it failed to address issues of self-determination, and has been ratified by only a small number of states not including Australia.

### 2.4. The Declaration

After more than 20 years in the making, the UNDRIP was finally adopted by the General Assembly in 2007 [59]. As the first human rights instrument acknowledging the collective rights of Indigenous peoples, the UNDRIP set out general principles and recognised rights to nationality, self-determination, equality and freedom from adverse discrimination; culture, spirituality, education and language; land and resources and participatory rights in development and other economic and social rights [59] (arts 1–6). While the UNDRIP is aspirational in nature and creates no enforceable rights in international law, it provides a framework for political advocacy and an authoritative statement on potentially emerging law concerning Indigenous people. Its value lies in the potential to crystallise new norms and practices, forming an emerging binding body of customary law [60] (p. 21).

In recognising the specific rights of Indigenous peoples, including collective rights and self-determination, it has challenged the capacity of the human rights enterprise to cope with diversity [28] (p. 375). Patrick Thornberry states that there is “no more ‘radical’ document in the field of international human rights [28] (p. 375). After being one of only four nations that voted against adoption of the UNDRIP in the General Assembly, Australia has since expressed its support in 2009 [61]. While there exists some contention about the fact that the development of the UNDRIP involved only a select few Aboriginal and Torres Strait Islanders, the Aboriginal and Torres Strait Islander Social Justice Commissioner has said that it remains one of the most significant milestones in the protection of Indigenous human rights [62]”.

Through the collective effort of Indigenous peoples across the globe, a specific set of rights and legal standards have now been established within the existing international framework that apply in addition to all other human rights. Indigenous peoples have, over some time, carved out a space in which the boundaries and founding principles of international law have been stretched and moulded to encompass their unique needs. Anaya and Kymlicka suggest that the inclusion of Indigenous rights in the international human rights framework demonstrates the emergence of a multicultural model of political ordering that challenges Western conceptions of the culturally homogenous and legally monolithic state [1] (p. 15), [63] (p. 158), [64]. In this way, it has potentially opened up the opportunity for legal structures to recognise and reflect on difference “not as otherness, or exclusive opposition, but rather as specificity, variation and heterogeneity [24] (p. 130), [65]”. In the following section, I explore more fully the arguments for and against the use of the human rights framework by Indigenous people.

## 3. Post-Colonial Discourses—Moving beyond the Dichotomy

The expanding compendium of postcolonial theory, and its application to human rights and Indigenous peoples, provides a lens through which to understand the shifts that have taken place with regards to international law and its application in the Indigenous context. By moving beyond fixed ideas of difference as otherness to foreground the fluidity of culture and diversity of society, as Homi Bhabha suggests [66], we can also come to understand the changing nature of law as a reflection of culture and society. Indigenous peoples’ use of and influence on a system of law that once justified their disenfranchisement is a working example of this [28].

Postcolonial theory draws our attention to the ways in which knowledge and power have been used to assert the superiority of one culture over another [23], [67] (p. 408), [68] (p. 175). It works to remind us of the way in which global inequalities are perpetuated through modes of representation and distribution of resources [69] (p. 250), and is important for framing innovative modes of resistance and recovery. Understanding the reciprocal relationships between domination, resistance and difference, as Said outlines, is important to undermining Eurocentric ontologies and recognising the open-ended influence of neo-colonial ideologies and practices. However, Fazal Rizvi et al. critically suggest that in focusing on difference as the mode of resistance, “Said ironically espouses a form of western humanism that accepts as unproblematic a secularist cosmopolitan world-view grounded in western enlightenment philosophies [69] (p. 253)”. In order to break out of dualistic conceptions of difference, theorists such as Bhabha, instead, argue that breaking the logic of binarism is the only way to destabilise the fixed position of a racialised dichotomy and the structures it re-inscribes [66], [70] (p. 220).

Moving beyond the dichotomy of Indigenous vs. non-Indigenous requires acknowledging that we live in a hybrid world where cultural boundaries are permeable and the definition of Indigeneity is not frozen in a fixed category of being, as asserted by imperial thinking [71] (p. 361). Bhabha argues that “cultural identities cannot be ascribed to pre-given, irreducible, scripted, ahistorical cultural traits [69] (p. 253)”. He builds on the work of Frantz Fanon, who said that neither the coloniser nor the colonised can be “viewed as separate identities that define themselves independently [72] (p. 253)”. Instead, cultural identity involves a continual exchange. That is, cultural boundaries do not exist *a priori*, but are a product of articulation between different elements of experience and subjective position [66], [73] (p. 225). Resistance to ongoing colonial power, as Bhabha affirms, can be achieved through the process of disrupting the exclusionary logics upon which discourses of colonialism, nationalism and patriarchy depend [66] (p. 45). This change of perspective enables a form of subversion “that turns the discursive conditions of dominance into the grounds of intervention [66] (p. 45)”. The emergence of the Indigenous voice in international law through the human rights discourse is one such example in which contemporary understandings of the postcolonial reposition colonisation as part of continuing transnational and transcultural processes, “exposing and rewriting earlier assumptions of nation-centred, imperial grand narratives, and reconfiguring a new field of discourse able to provide tools with which to think about the present [74] (p. 824)”.

The risk of this approach, as already suggested, is that the colonised subject’s mode of resistance is itself constrained by the nature of the tools being utilized. Wendy Brown, in her exploration of the paradox of rights with regard to the promise of women’s equality, suggests that a problem arises when rights only work to “build a fence” around the identity of the human rights victim at the site of violation, “regulating rather than challenging the conditions within [75] (p. 231), [76]”. While hybridity is a useful antidote to the long history of European cultural essentialism, it can also run the risk of further embedding or legitimating dominant structures [77] (p. 109), [78] (p. 46). That is, rights can be seen simultaneously as proffering a path to freedom and delineating the boundaries within which that freedom is found. As Shohat and Stam warn, “(a) celebration of syncretism and hybridity per se, if not articulated with the issues of hegemony and neo-colonial power relations, always runs the risk of appearing to sanctify the fait accompli of colonial violence [79] (p. 213)”. Proponents of the universal vs. relativism debate contend with these same issues. How can we illuminate the hidden assertions of natural law and make use of the idea of universal standards? 

Sally Engle Merry sees that by reframing culture as something that is “contested, historically changing, and subject to redefinition”, it is also possible to reimagine the relationship between culture and law [38] (p. 602).

*Focusing on production and appropriation provides a framework that recognizes the agency of subordinated peoples at the same time as it emphasizes the political and economic constraints on that agency. It replaces ideas of imposition with an analysis of the negotiated and partial nature of transformation*.

*[38] (p. 580)*

While some scholars see resistance in dualistic terms, as a clash between dominant and subordinate groups, Merry sees that resistance can also take the form of cultural appropriation [38] (p. 601). The concept of a subordinate group utilising existing structures to their benefit, she submits, provides a “way of understanding social transformation that is attentive to agency, to competing cultural logics, and to the complexity of the social fields within which change takes place [38] (p. 602)”. It incorporates the possibility of resistance, while recognising that resistant practices may involve actions that appear to be accommodation and adaption [38] (p. 602). The distinguishing feature is that such resistance restructures the positioning of the subordinate group within the hegemonic structure of the dominant society [38] (p. 602). “The underlying dichotomy inherent in western liberalism is not forgotten or unrecognized, but accepted with the future aim of forging a renewed identity within it [66]”.

Culture, Merry asserts, is integral to systems of power and law, and if culture is understood as a “flexible repertoire of practices and discourses that are created through historical processes”, then law must also be understood as being in a continual state of transformation, rather than homogenous, static or embedded. In this way, the plurality of law, its transformative property, makes it possible for it to accommodate and provide leverage for modes of resistance [38] (p. 603). Indigenous peoples’ use of, and influence on, human rights law and discourse provides a good example of this process. What can be seen is the transformation of a system of law as a result of continuing and multiple acts of resistance. Indigenous peoples have, in the absence of any effective alternative, worked within the prevailing legal system to reposition themselves as makers of law and creators of change. This process of accommodation and adaptation has meant that the law itself has been contested and transformed so that a space for collective rights has been opened in the liberal framework of individual human rights. A system embedded in dichotomies of difference has been upended to reflect accommodations of difference and acceptance of multiplicity.

With regards to Indigenous health in Australia, the impact of this act of appropriation at the international level can be seen as filtering down to the domestic level through the increasing use and implementation of the human rights discourse in Aboriginal and Torres Strait Islander health policy, as well as filtering up, with local practices used to inform international developments. In the following section of the paper, I further explore the relationship between Indigenous health and human rights by examining the emergence of a rights-based approach to improve Aboriginal and Torres Strait Islander health outcomes. 

## 4. Human Rights and Aboriginal and Torres Strait Islander Health 

The use of, and engagement with, the human rights framework by Indigenous peoples, as Anaya suggests, has been transformative:

*(it) advances, on the one hand, cultural integrity and autonomy and, on the other, participatory engagement. This dual thrust reflects the view that Indigenous peoples are entitled to be different but are not necessarily to be considered a priori unconnected from larger social and political structures*.

*[21] (p. 60)*

The relationship between Aboriginal and Torres Strait Islander peoples and the right to health at international law provides an instructive example of how an Indigenous social movement appropriated and influenced the human rights framework to their mutual benefits. In this section, I examine how human rights provided an international platform and language with which to reinforce an already strong positioning within the Indigenous-led movement that argued for a better understanding of four key components that were eventually included in the national Indigenous health policy: the impact of social determinants on health; the need for specialised rights supported by notions of equality of opportunity; the importance of governmental responsibility to ensure equal enjoyment of rights to health; and the need for community participation in the design and delivery of comprehensive health care models.

The foregrounding of important links between health and human rights by Aboriginal Community Controlled Health Organisations (ACCHOs) during the 1990s has had a significant impact on elevating the importance of community control within the international human rights framework, and they have been recognised as a model of best practice for the implementation of the right to self-determination as enshrined in the UNDRIP [80] (p. 11). The human rights framework, in turn, has provided Indigenous peoples with an external framework or set of tools with which to support their own initiatives and continue to influence state behaviour.

### 4.1. Linking Human Rights and Indigenous Health

While Indigenous peoples of Australia were arguing for the recognition of specialised rights in international human rights law, they were also utilising the existing human rights framework in the domestic setting to advance local causes. As stated earlier, the rights-based approach was adopted to bolster support for civil, political and land rights and was also being applied to campaigns and specialised initiatives to improve Indigenous health. As a human right, applicable to all, the right to health at international law offered a range of principles that resonated with the Aboriginal and Torres Strait Islander approach to influencing change, although governments have been slow to respond. While references to the right to health were present in early World Health Organization (WHO) documents, the active link between health and human rights only gained widespread traction in the international public health discourse of the 1990s, in response to the HIV AIDS crisis in Africa [81,82,83]. Yet, the association between Indigenous health and the rights-based framework is evident much earlier in the Indigenous-specific programs delivered by Aboriginal community-controlled cooperatives and health services in the early 1970s [16], and the National Strategy for Aboriginal Health of 1989 (although it was found that this strategy was never properly implemented) [84,85] (p. 3). 

First articulated in the international context in the WHO Constitution of 1946, health was defined as “a state of complete physical, mental and social well-being and not merely the absence of disease or infirmity [86]”. The WHO recognised that the “enjoyment of the highest attainable standard of health is one of the fundamental rights of every human being without distinction of race, religion, political belief, economic or social condition [86]”. By characterising health holistically, as more than just physical health, the WHO acknowledged the central impact of social determinants on human health and well-being [87], and by highlighting health as a fundamental right, health was elevated to a significant consideration in the international context. 

The UDHR embedded this broad approach by including health as a component of the right to an adequate standard of living in Article 25 [88]. Following the lead of the WHO, the ICESCR recognised “the right of everyone to the enjoyment of the highest attainable standard of physical and mental health” in Article 12, listing some of the health-related legal obligations of ratifying states [89]. The WHO reaffirmed its earlier definition of health in the Alma Ata Declaration of 1978:

*The Conference strongly reaffirms that health, which is a state of complete physical, mental and social wellbeing, and not merely the absence of disease or infirmity, is a fundamental human right and that the attainment of the highest possible level of health is a most important world-wide social goal whose realization requires the action of many other social and economic sectors in addition to the health sector*.

*[90]*

The definition of health, as outlined in these human rights treaties and interpretive instruments, corresponded with the notion of health as expressed by Aboriginal and Torres Strait Islander people, in which health is seen in a holistic context, encompassing physical health alongside spiritual, cultural, emotional and social well-being [91] (p. 4), [92] (p. 128). Indigenous Australians have historically emphasised that their health is linked to their ability to collectively control their lives and cultures, and to the recognition of their political, civil, cultural, social and economic rights [93] (p. 17). In 1989 the first national strategy on Aboriginal health was developed through a comprehensive and inclusive national consultation process with Indigenous Australians. The resulting definition of health, set out as follows, mirrored many of the principles outlined by the WHO and the ICESCR:

*“Aboriginal (and Torres Strait Islander) health” means… not just the physical wellbeing of an individual but refers to the social, emotional and cultural wellbeing of the whole community. This is a whole-of-life view and it also includes the cyclical concept of life-death-life*.

*[84] (p. 5)*

*“Health” to Aboriginal (and Torres Strait Islander) peoples is a matter of determining all aspects of their life, including control over their physical environment of dignity, of community self-esteem, and of justice. It is not merely a matter of the provision of doctors, hospitals, medicines or the absence of disease and incapacity*.

*[84] (p. 4)*

Reflecting and reiterating parts of the Declaration of Alma-Ata, the Strategy gave support to existing domestic arrangements for community participation in the design and delivery of primary health care, defining care as:

*Essential health care based on practical, scientifically sound, socially and culturally acceptable methods and technology, and universally accessible to individuals and families in the communities in which they live through their full participation at every stage of development in the spirit of self-reliance and self-determination*.

*[84] (p. 5)*

The strategy also made specific reference to the role of the UN in influencing governments to adequately resource Aboriginal health and appealed to definitions of community participation as set out by the WHO’s “Formulating Strategies for Health for All by the year 2000 [84] (p. 16)”.

Laying the groundwork for these important initiatives was the establishment of the first Aboriginal health service in Redfern, in Sydney, in 1971, which aimed to provide accessible health services for the increasing and largely medically uninsured Aboriginal population [94]. The Redfern health service was well in advance of international aspirations at the time, with its commitment to developing an “accessible, effective, appropriate, needs-based health care” with a focus on prevention and social justice [94]. By 1978 there were 12 Aboriginal Community Controlled Health Services across the country funded in various ways, including by donation, loans from staff and through assistance from the public health system (Medibank) and international aid agencies [95]. Numbering around 140 in 2018, services maintain the foundational aims and are initiated and operated by the local Aboriginal community to deliver “holistic, comprehensive, and culturally appropriate health care to the community which controls it, through a locally elected Board of Management [96]”. This allows Aboriginal communities to determine their own affairs, protocols and procedures [96] and has made way for a model, in keeping with a holistic view of health, in which medical care and treatment is offered in conjunction with social, mental health and financial services. While funding for these services is still dependent on government, private support of these services is increasing. 

Broader academic debate and public interest in the practical application of human rights to public health caught up with what was happening in the Aboriginal community-controlled health sector throughout the 1990s. The active positioning of health within a human rights framework spearheaded an important shift in ways of analysing and responding to public health challenges, which complemented existing initiatives in Aboriginal and Torres Strait Islander health [87] (p. 924). International recognition that public health and human rights paradigms were concomitant approaches to defining and advancing human well-being were far more useful (as suggested by Farmer, Mann and others) than relying solely on a biomedical- and pathology-based approach [81], [82] (p. 9). This recognition opened up “new tools for challenging and reimagining the utilitarian and technical approaches to health” and contested entrenched views of prevailing health standards and practices [97] (p. 275). By embedding the right to health in international law, Dianne Otto argues, health became a legal entitlement rather than a “privilege, commodity or result of altruism [97] (p. 276)”. Societies (and their governments) are obliged then to recognise their roles and responsibilities and to take action and change the “conditions which constrain health and create vulnerability to preventable disease, disability and premature death [98] (p. 230)”.

This expanded view of health, linking social justice to the correction of health disparities, provided the possibility of simultaneously addressing the societal causes as well as their pathological expressions [98] (p. 232), [99] (p. 95). Health came to be properly understood in the international context, in terms of a continuum, with the enjoyment of civil and political rights, as well as economic and social rights, as essential elements to promoting and protecting the well-being of individuals and the collective [83] (p. 1493), [100].

Further, the interconnected nature of human rights and the principles of equality and non-discrimination supported the need to recognise the specificity of health standards as they apply to particular groups, especially where the health of those groups is “affected by their positions of subjugation [81], [97] (p. 277)”, [101,102]. In the absence of any strong domestic levers, the right to health, as articulated in the human rights framework, provided Indigenous peoples with an external framework or set of tools with which to support their own initiatives to influence state policy and practice [12].

The WHO and the UN Human Rights Council (formerly the Commission on Human Rights) have paid increasing attention to the right to health, and human rights treaty bodies have adopted general comments or general recommendations on the right to health and health-related issues [103] (p. 2). In 2002, the UN Special Rapporteur on the right of everyone to the highest attainable standard of physical and mental health was appointed to clarify the nature of the right and how it can be achieved [104]. The right has also been affirmed and expanded over time in its application to discrete groups protected by other human rights treaties [105]. The right to health, as expressed in the ICESCR, was reflected in the UNDRIP, which states that “Indigenous individuals have an equal right to the enjoyment of the highest attainable standard of physical and mental health [59] (art. 24(2))”. The UNDRIP also strongly emphasises the importance of social determinants in improving health, and the right of Indigenous peoples to take an active part in decision-making processes in matters that affect their rights as well as administering those programmes through their own institutions [59] (arts. 17–19, 23).

What can be seen from this historical overview is that health provides an important site of coalescence in which the philosophical approaches to health championed by the Indigenous community complement initiatives in the international arena and vice versa. Megan Davis, who served as Australia’s expert member on the UN Permanent Forum on Indigenous Peoples, claims that input from the National Aboriginal Community Controlled Health Organisation has made “a very real difference to the health mandate” of the Forum [80] (p. 11), especially in their advocacy for community control. She contends that the community-controlled model of health is a best practice example of the implementation of the right to self-determination:

*The community control sector—more than any other sector—deal with the bread and butter of self-determination—choices people make about their lives each and every day. It is very apparent to me that the health community control sector is implementing the UNDRIP in terms of leading the way on the right to self-determination—what it looks like in practice*.

*[80] (p. 13)*

This holistic approach to self-determination represents a departure from earlier campaigns in which arguments for self-determination were couched in terms of a separation from settler society and advancing a model of discrete governance. What Davis expresses here is analogous with Merry’s view of agency in which Aboriginal and Torres Strait Islanders claim authority in their own affairs through designing and actively producing services that are suitable for them, and by actively taking advantage of the opportunities that are present within an existing framework [106] (p. 15), [107,108]. It is a political vision that is neither assimilationist nor separatist, but one that is better aligned with Bhabha’s view of a hybrid society. It is an approach in which social negotiations of difference are understood to involve considerable exchange, interaction and interdependency, and whereby the heterogeneous nature of the polity is consciously recognized and embraced [24] (p. 135).

### 4.2. Implementation

There is ongoing debate about the value and impact of human rights at a local level and, in particular, for Indigenous peoples as they are dependent on the state acting as an agent of social change [83] (p. 1493). While states can ratify human rights treaties, in Australia it is not until those rights are formalised through the incorporation of those principles into domestic constitutional or statutory law that there is any effective legal mechanism to enforce those rights. Some suggest that without legal implementation, a focus on human rights is all but futile [109]. Others, however, argue that the substantive influence or the moral imperatives created by human rights is important and that their influence can be seen in subsequent government policies, decisions and actions [80] (p. 11), [110] (p. 45), [111]. In this section, I will look at the practical ways in which the human rights framework is being implemented in Australia with regards to Indigenous health. The remedial regime that is being influenced by international doctrine and domestic activism is one that focuses on context-specific arrangements to ensure the effective involvement of Aboriginal and Torres Strait Islanders within the context of the Australian state.

#### Indigenous Health and Human Rights in Australian Policy

In Australia, legislative implementation of international treaties has a relatively poor history [109] (p. 449), [112]. Having regard to the more substantive indicators of implementation is, therefore, important for gauging the influence and relevance of international human rights law in the domestic setting, especially with regards to the right to health for Aboriginal and Torres Strait Islanders [110] (p. 50). The links between health and human rights, and their influence on debates over health policy, health service delivery, and development policy, is being increasingly recognised globally [101] (p. 1), [103] (p. 2). As Harrington and Stuttaford observe:

*The human right to health has moved to the centre of political debate and social policy across the globe. Civil society organizations have put this right at the heart of campaigns for health justice at national and global levels*.

*[113] (p. 1)*

John Tobin is more sceptical about the centrality of the role of rights in transforming domestic policy, suggesting that the right to health is much less secure and far more marginalized. In Australia, he asserts, although policy makers may have accepted the linkage between health and human rights, he sees little expression of the right in National health care reforms [103] (pp. 2–4). He does concede, however, that for those states that have accepted an obligation to act in good faith and ensure the effective implementation of the right to health, it can have immense transformative and deeply political potential:

*It demands an evaluation of the cultural and social practices, systems, and structures, both local and global, which undermine the availability, accessibility, acceptability, and quality of such medical services and the social determinants of health. And it demands a commitment from states to take reasonable measures to transform these practices, systems, and structures to secure the right of every individual to the highest attainable standard of physical and mental health*.

*[103] (p. 375)*

While there are few legal sanctions to compel states to implement their human rights obligations, they are increasingly monitored for their compliance with human rights norms by human rights treaty bodies, other states, nongovernmental organisations, the media and private individuals [81] (p. 11), [114].

While the right to health has not been formally implemented into legislation in any comprehensive way in Australia, with regards to Indigenous health, its influence is acknowledged in a number of policy initiatives including and following the 1989 National Aboriginal Health Strategy [84,115,116]. Importantly, in response to the ongoing poor health outcomes for Indigenous Australians, Tom Calma, the then Australian Human Rights Commission’s (HREOC) Aboriginal and Torres Strait Islander Commissioner, demanded that the Australian Government adopt a human rights-based approach to improving Indigenous health. In his Social Justice Report of 2005, he characterised Indigenous health inequality as a major human rights issue [117]. The report provided the foundation for HREOC’s Close the Gap Campaign for Indigenous Health Equality, mobilising support from a range of health and human rights organisations and inviting personal pledges from the Australian public to help raise the life expectancy and improve the health of Indigenous Australians so as to achieve equality with non-Indigenous Australians within a generation.

Calma asserted that the major failing of past Australian government initiatives is that they had not properly “activated” their commitments to Indigenous health equality by setting time-bound targets or benchmarks, with commitments for the necessary funds and program supports for success [12] (p. 22).

*Ultimately, human rights standards provide a system to guide policy making and to influence the design, delivery and monitoring and evaluation of health programs and services. It is a system for ensuring the accountability of governments*.

*[12] (p. 48)*

Importantly, he drew on some major aspects of international law and a human rights-based approach to support the development and design of these implementation measures. These are the overarching principles of non-discrimination and progressive realisation in the context of health; the role of participatory processes in realising human right standards; and acting comprehensively on all aspects of the content of the right to health [12] (p. 26), [118], which I explore in more details below.

Non-discrimination. The non-discrimination principle, outlined in Article 2(2) of ICESCR [119], is a cornerstone of human rights, and entitles every person to be treated equally and to substantively enjoy their rights without discrimination. It requires states that have ratified this and other relevant treaties, such as the CERD, to take immediate steps to actively redress inequality in the enjoyment of rights, such as the right to health [118,120]. These steps might involve “special measures” that require differential treatment that is considered non-discriminatory because its goal is to achieve substantive equality [12] (p. 27). In the context of Indigenous health, this ensures that health infrastructure and services are designed to accommodate difference and works to address issues of structural racism that exists within the health system [118]. It means that there should be no universal approach to health, but a hybrid system that is responsive to, and inclusive of, Indigenous culture in its many manifestations.

Progressive realisation. Progressive realisation, or the obligation to “take steps” towards full realisation of ICESCR rights, as set out in Article 2(1), requires that governments take deliberate, concrete and targeted steps, as soon as possible, to ensure that everyone fully enjoys the relevant rights [121]. This includes monitoring progress by identifying appropriate indicators and benchmarks that are specific, time bound and verifiable [12] (p. 27). For Indigenous health policy, which has historically been devoid of targets and accountability mechanisms [122], it provides an important impetus for governments to ensure that the measures they implement have the appropriate financial backing to be successful; that progress is regularly monitored; and that strategies are designed to be effective and achievable.

Participation in Decision Making. The Declaration of Alma-Ata promoted the principle that communities should contribute to the development of policies and programmes that affect their health [90]. The Committee on Economic, Social, and Cultural Rights built on this in General Comment 14, adopted in 2000, whereby it sought to better define the right to health (as set out in Article 12, and reaffirmed in Article 24(2) of the UNDRIP [59]). General Comment 14 stated that the right to health of Indigenous peoples would include:

1. Greater involvement of Indigenous peoples in decision-making processes that affect them;
2. Greater control over the administration of Indigenous health programs;
3. Emphasis on culturally appropriate services;
4. Recognition of traditional healing methods;
5. Emphasis on the social determinants of health;
6. Coordination of policies across departments and levels of government; and
7. Emphasis on equality in the provision of health services [110] (p. 51), [123].

As noted throughout the paper, participatory processes have been at the core of the Aboriginal community-controlled healthcare sector since its inauguration in 1971, and are an important part of the UNDRIP [59] (arts. 18, 19, 23). Participatory processes are integral to the ideas of agency and central to theories of cultural appropriation in which Indigenous peoples regain control over the decisions that are made about their lives [38] (p. 585).

Acting on the Content of the Right to Health. In General Comment 14, the Committee on Economic, Social, and Cultural Rights interpreted the right to health as containing the four interrelated and essential elements: making public health and healthcare facilities, goods and services readily available; ensuring their acceptability to a diverse range of individuals and communities; ensuring they are accessible and free from discrimination; and are of good quality [123] (para. 12). The important aspect of the right to health as enshrined in human rights law, notes Tobin, is that it is “not a right to be healthy but an entitlement to receive and enjoy the services, facilities, and conditions that are necessary to prevent, remedy, and mitigate ill health [103] (p. 173)”. Implementing the right therefore includes creating social conditions that offer everyone an equal opportunity to be healthy. In reference to Indigenous people, the Committee advised governments to implement special measures to advance Indigenous health and provide resources for Indigenous peoples to design, deliver and control culturally appropriate services so that they may enjoy the highest attainable standard of physical and mental health [123] (para. 27).

According to this overall approach, an Indigenous worldview and perspective is central to the development, implementation, and evaluation of programs and interventions and represents the application of human rights in the name of improvements for Indigenous people [124] (p. 111). The campaign that Calma initiated represented a multilevel approach to Indigenous health requiring the involvement of policy-makers, organisations and community members in actively integrating human rights principles, standards and commitments into national programs. To date, 200,000 Australians have pledged their support for the Close the Gap Campaign goals, and an annual National Close the Gap Day attracts over 140,000 people to around 850 events each year [125] (p. 1).

Following the election of the Australian Labor Government in 2007, and as a result of the ground swell of support from individuals and Indigenous and non-Indigenous organisations, many aspects of HREOC’s Close the Gap Campaign became official government policy in the form of the Close the Gap Statement of Intent [126,127] signed by both major parties (and later by most State and Territory Governments and leaders of Opposition), signalling a clear set of commitments and a whole-of-government approach to improve Indigenous health by 2030 [127]. The Statement provided a framework that set coherent targets for achieving change, was inclusive of the social and cultural determinants of health and committed the government to working in partnership with Indigenous Australians to design and deliver services that are suitable [126]. Whilst the subsequent Closing the Gap initiatives stemming from the Council of Australian Governments (COAG) fall short of the human rights-based approach championed by the Close the Gap Campaign [128], the Statement of Intent was foundational in making the aim of achieving Aboriginal and Torres Strait Islander health equality within a generation a national priority and contributed as step forward in supporting a more holistic and participatory approach to Indigenous health as well as setting targets to achieve outcomes. 

In 2013, the emphasis on a human rights-based approach was embedded in earnest through the National Aboriginal and Torres Strait Islander Health Plan 2013–2023 [116] that was developed in partnership with Aboriginal and Torres Strait Islander peoples through the National Health Leadership Forum. The Health Plan highlights Health Equality and a Human Rights Approach as one of its overarching principles and directly refers to the value of UNDRIP and other human rights instruments in supporting the improvement of Aboriginal and Torres Strait Islander Peoples’ health. The Close the Gap Steering Committee for Indigenous Health Equality reports that:

*The development of the Health Plan demonstrates that the capital in the knowledge (including cultural knowledge), leadership and lived experience of this leadership group should not be underestimated. In particular, the emphasis in the Health Plan on the importance of social and emotional wellbeing; culture; and the need to address racism as a negative social determinant of Aboriginal and Torres Strait Islander health; can be identified as unique contributions of the (Forum) to this key strategic document in the national effort to close the gap*.

*[125] (p. 14)*

In addition to the Health Plan, the Australian government launched the National Aboriginal and Torres Strait Islander Suicide Prevention Strategy [129], a commitment to renew the Social and Emotional Wellbeing Framework [130] and the National Partnership Agreement on Indigenous Early Childhood Development [131]. These provide opportunities to progress important areas of Aboriginal and Torres Strait Islander mental health, social and emotional wellbeing and education, alongside the implementation of the Health Plan.

Importantly, these initiatives prioritise Aboriginal Community Controlled Health Services and their human rights-based approach as an integral part of improving health systems for Indigenous Australians. While the impact on overall health outcomes is yet to be significantly recorded, a result of the complex interplay of a range of social and economic factors, Kathryn Panaretto and her colleagues claim that the focus in Community Controlled Health Services on prevention, early intervention and comprehensive care has reduced barriers to access and unintentional racism, and is progressively working to improve individual health outcomes for Aboriginal people [132] (p. 649). Combined with strategies targeting housing, lateral violence, education and employment, this comprehensive approach provides an important foundation for improving health outcomes.

Efforts to embed a human rights-based approach to improving the health and life expectancy of Aboriginal and Torres Strait Islander people have represented a multi-decade commitment spanning policy cycles, funding agreements and governments [128] (p. 2) and is the culmination of an Indigenous led movement that influenced and then utilised the human rights framework in an effort to improve health outcomes of Aboriginal and Torres Strait Islander people at the domestic level. The content on the right to health not only reflected the already existing principles of the Aboriginal and Torres Strait Islander health movement, but provided a set of legal, moral and political arguments with which to further initiatives that addressed Indigenous marginalisation and built capacity in the health sector. Human rights have provided communities with a language that governments can understand and respond to. In the case of the UNDRIP, in particular, it is a language embedded with Indigenous voices. Whilst the application of a human rights-based approach has not yet achieved the desired outcome for Indigenous Australians to be as healthy as their non-Indigenous counterparts, human rights, in this context, can be seen to strengthen capacities, promote productive cultural hybridities and empower innovative processes that operate within and beyond existing institutional systems and structures.

## 5. Conclusions

In the face of devastating social, political and economic conditions, Aboriginal and Torres Strait Islanders have sought not only to resist and react, but to rebuild and reimagine their cultures as well as the political and legal constructs that have been imposed on them [133] (p. 21). In doing so, the view of Indigenous peoples as passive objects in a Eurocentric story of historical progress has been replaced with Indigenous viewpoints that can be seen in constant interaction and engagement with other cultures and the law [133] (p. 21). Postcolonial interpretations of hybridity and multiplicity reveal Indigenous/non-Indigenous dichotomies to be fluid and contested constructs, momentary locations of “past and present, inclusion and exclusion, difference and similarity [74] (p. 833)”. These insights allow us to reject fixed notions of culture, politics and law and go to the heart of what Williams describes as the “alchemy” of rights [134]. In the context of health, “breathing life” [135] (p. 163) into the human rights framework, by having an impact on it and utilising it in local contexts, has provided Aboriginal and Torres Strait Islanders with a powerful set of moral, political and legal arguments with which to influence change. As Williams states, rights are “the magic wand of visibility and invisibility, of inclusion and exclusion, of power and no power. The concept of rights… is the marker of our citizenship, our relation to others [135] (p. 164)”. While we must remain cognisant of what Gayatri Spivak deems the “dilemma” of rights [75] (p. 230), [76] (p. 451), [78] (pp. 45–46), in which the path to freedom is delineated by the legal boundaries within which that freedom is found, for Aboriginal and Torres Strait Islanders, asserting a place of influence in the decision-making processes of the existing frameworks with established mechanisms has been an essential component of enacting and securing agency. Appropriating and redefining the mechanism of international law has consequently emphasised the plurality of culture and the malleability of law and has led to improved national policy initiatives [38] (p. 602).

Indigenous peoples’ efforts to engage with the human rights framework, despite its potential dangers, has resulted in significant legal and political change. While the human rights framework has limitations, its expansion represents a slow but important global move toward unravelling entrenched hierarchies of difference and is a working example of how community led social movements have influenced radical changes within an imposed system of law. Indigenous peoples and their organisations are now recognised as significant participants in the framing of international human rights norms [134] (p. 186), and this has, and will continue to have, increasing impact on national policy and law. With regard to Aboriginal and Torres Strait Islander health, the synergy between the rights-based approach and Indigenous led initiatives has elevated the importance of the social determinants of health, the need for specialised rights and for community participation in the design and delivery of comprehensive health care models.
